# Distinct patterns of blood-stage parasite antigens detected by plasma IgG subclasses from individuals with different level of exposure to *Plasmodium falciparum *infections

**DOI:** 10.1186/1475-2875-9-296

**Published:** 2010-10-26

**Authors:** Cathrine Holm Olesen, Karima Brahimi, Brian Vandahl, Susana Lousada-Dietrich, Prajakta S Jogdand, Lasse S Vestergaard, Daniel Dodoo, Peter Højrup, Michael Christiansen, Severin Olesen Larsen, Subhash Singh, Michael Theisen

**Affiliations:** 1 Department of Clinical Biochemistry, Statens Serum Institut, Copenhagen, Denmark; 2 Department of Immunology, Infectious Disease Immunology, Statens Serum Institut, Copenhagen, Denmark; 3 Centre for Medical Parasitology at Department of International Health, Immunology and Microbiology, University of Copenhagen, Copenhagen, Denmark; 4 Parasitology Laboratory at the Department of Bacteriology, Mycology and Parasitology, Statens Serum Institut, Copenhagen, Denmark; 5 Noguchi Memorial Institute for Medical Research, University of Ghana, Legon, Ghana; 6 Department of Biochemistry and Molecular Biology, University of Southern Denmark, Denmark; 7 Bio-Medical Parasitology Unit, Pasteur Institute, Paris, France; 8 Indian Institute of Integrative Medicine, Jammu, India; 9 Novo Nordisk A/S, 2820 Gentofte, Denmark

## Abstract

**Background:**

In endemic regions naturally acquired immunity against *Plasmodium falciparum *develops as a function of age and exposure to parasite infections and is known to be mediated by IgG. The targets of protective antibodies remain to be fully defined. Several immunoepidemiological studies have indicated an association of cytophilic anti-parasite IgG with protection against malaria. It has been hypothesized that the initial antibody responses against parasite antigens upon first few *Plasmodium falciparum *infections is dominated by non-protective IgG2/IgG4 and IgM antibodies, which then gradually develop into protective response dominated by cytophilic IgG1 and IgG3 antibodies.

**Methods:**

Naturally occurring IgG antibodies against *P. falciparum *blood-stage antigens were analysed from plasma samples collected from four groups of individuals differing in age and level of exposure to *P. falciparum *infections. Western Blot profiling of blood-stage parasite antigens displaying reactivity with individual plasma samples in terms of their subclass specificities was conducted. Parasite antigens detected by IgG were grouped based on their apparent molecular sizes resolved by SDS-PAGE as high molecular weight (≥ 70 kDa) or low molecular weight (< 70 kDa). The number of discernable low molecular weight parasite antigens detected by different IgG subclass antibodies from each plasma sample was recorded. Using Wilcoxons rank sum test these reactivities were compared amongst groups of individuals with different levels of exposure to *P. falciparum *infections.

**Results:**

IgG4 and IgM antibodies in plasma samples from all groups detected very few parasite antigens. IgG2 antibodies from all groups detected a common pattern of high molecular weight parasite antigens. Cytophilic IgG subclasses in plasma samples from individuals with higher levels of exposure to *P. falciparum *infections distinctly detected higher numbers of low molecular weight parasite antigens.

**Conclusions:**

In the present study, there was no evidence for switching of antibody responses from non-cytophilic to cytophilic subclasses against blood-stage parasite antigens as a likely mechanism for induction of protective immunity against malaria.

## Background

Immunoepidemiological studies have demonstrated that immunity against blood stage *Plasmodium falciparum *is associated with the acquisition of anti-parasite antibodies of the cytophilic subclasses [1], and in particular IgG3 [2-9]. No such protective association has been observed for non-cytophilic subclasses such as IgM and IgG4 [2,3]. For IgG2 conflicting evidence has been presented, associating levels of specific IgG2 antibodies with either an increased frequency of clinical malaria episodes [1,2,10], or resistance to *P. falciparum *malaria [11,12]. It is noteworthy that protection against malaria by IgG2 has often been associated with the FcγRIIa-H131 allotype, a receptor point mutation which accords binding to IgG2 [11,13-16]. These observations support the importance of cytophilic antibodies in protection against malaria. It has been hypothesized that development of effective IgG-mediated anti-parasite immunity depends on the maturation of antibody responses, not only in terms of their antigen specificities and affinity maturation, but also in terms of class-switching implying that the progressive development of malaria immunity in older children can be attributed to a switch of anti-parasite antibodies from the non-cytophilic to the cytophilic subclasses [3,17]. It has even been proposed that the non-cytophilic antibodies could compete and block the protective mechanisms elicited through the binding of the cytophilic subclasses [17].

The subclass profile of naturally occurring IgG responses has therefore been extensively studied for several major blood-stage malaria vaccine candidate antigens. These analyses have mainly been carried out by ELISA using recombinant proteins or synthetic peptides usually representing subdomains of malarial proteins as test antigens. Such antigen preparations do not always accurately mimic native parasite protein conformations, including post-translational modifications. A more global approach was therefore adopted to study the targets of the naturally occurring anti-parasite IgG subclass responses through IgG subclass specific Western blot analysis of total parasite proteins expressed in mature blood stage schizonts. Purified Parasitophorous Vacuole Membrane-Enclosed Merozoite Structures (PEMS) [18] were used as a source for parasite antigens, because PEMS preparations i) contain a highly homogeneous synchronous parasite population at the mature schizont stage and ii) they are essentially free of contaminating host cell proteins.

Profiling of different naturally acquired IgG responses, in terms of their subclass specific recognition of parasite PEMS proteins, in individuals with different levels of exposure to *P. falciparum *infection is reported. Plasma samples were collected from four distinct sub-groups including: Group A: non-immune Danish travellers with a single episode of *P. falciparum *malaria; Group B: young (0-5 years) and Group C: older (6-10 years) Ghanaian children with frequent episodes of clinical malaria; and Group D: clinically immune Liberian adults. A group of non-immune Danish healthy adults (Group E) never exposed to malaria was included as control group.

## Methods

### Parasite cultures and purification of PEMS

*Plasmodium falciparum *(F32 strain) was cultured *in vitro *in human RBCs as previously described [19] using RPMI 1640 medium supplemented with 25 mM HEPES, 20 mM NaHCO_3_, 2 mg/liter hypoxanthine, 0.5% (w/v) AlbuMAX I, and 1% (v/v) penicillin-streptomycin at 5% v/v hematocrit. Cultures were synchronized by repeated treatment of parasites with D-sorbitol [20]. PEMS preparations were purified according to the protocol of Salmon *et al *[18]* *. Briefly, highly synchronized cultures at approx. 20% parasitemia were grown until the parasites had reached the schizont stage (> 8 nuclei stage), at which time they were treated with 10 μM E64 (Sigma, Germany) for 8-10 h until the PEMS emerged. These PEMS were brought into suspension, incubated for another 2 h to allow for the vast majority of RBCs to sediment and then purified from the supernatant by centrifugation through 60% v/v Percoll. PEMS were collected from the top of the cushion, centrifuged for 10 min at 2000 × g, washed three times in PBS containing protease inhibitor cocktail (Roche), and stored as packed cells at -80°C until use. The quality and homogeneity of the purified PEMS were routinely evaluated by light microscopy of Giemsa-stained smears and by SDS-PAGE. When adjusted for protein concentrations and under identical sample processing conditions, samples from different batches showed very similar protein patterns on Coomassie stained 1D-SDS-PAGE and silver stained 2-D gels (data not shown).

### Serum samples and ELISA

Plasma samples from Liberian adults clinically immune to malaria (n = 12), Danish donors never exposed to malaria (n = 35), and Ghanian children (n = 14) were selected randomly from previously published studies [21,22]. Plasma from non-immune Danish travelers (n = 7) were obtained from a routine diagnostic laboratory at SSI. These samples were from adults who i) had experienced a single attack of clinical *P. falciparum *malaria, and ii) had their malaria episode confirmed by a positive IFA test. Enzyme-linked immunosorbent assay (ELISA) was performed essentially as described [23] using an F32 late-stage schizont extract at a coating concentration of 50 μg/ml. All plasma samples were tested at a dilution of 1:200 in PBS, pH 7.0.

### SDS-PAGE and immunoblotting

One dimensional SDS-polyacrylamide gel electrophoresis (PAGE) and immunoblotting was carried out as per standard procedures described earlier [24]. Membranes were incubated with individual plasma samples diluted 1:200 in PBS, pH 7.4. For IgG subclass detection, monoclonal mouse anti-human subclasses IgG1 to IgG4 (clones NL16, HP6002, Zg4, and RJ4 [Skybio]) were used as secondary antibodies in dilutions of 1:1000 followed by incubation with alkaline phosphatase (AP)-conjugated rabbit anti-mouse IgG (Dako) at 1:1000.

### Statistical analysis

The differences between the distributions of parasite antigen bands recognized by plasma IgG from different individuals were compared by calculating two-sides P-values based on Wilcoxons rank sum test with correction for ties. The differences between ELISA values were compared by the u-test.

## Results

### Levels of naturally acquired anti-parasite IgG vary amongst individuals with different levels of exposure to parasite infection

**Figure 1:**
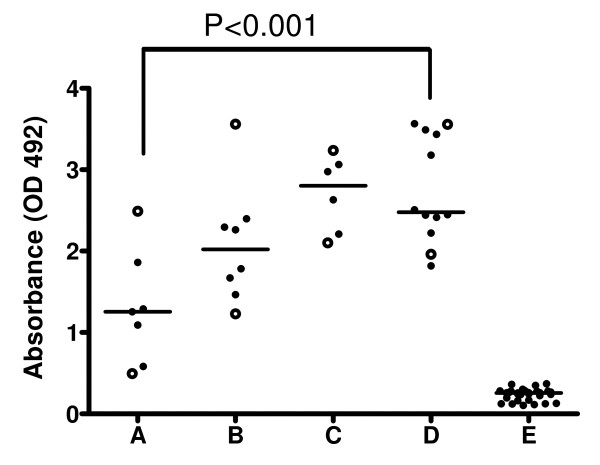
**ELISA titers against late-stage parasite extract**. Antibody levels in 7 non-immune Danish travellers with a single episode of clinical malaria (A), 8 Ghanaian children (0-5 years) (B), 6 Ghanaian children (6-10 years) (C), 12 Liberian adults clinically immune to malaria (D), and 35 non-immune Danish controls (E). Each point represents one plasma sample. OD492, optical density at 492 nm. The horizontal lines mark the mean value. Open circles mark the plasma samples for representative profiling of antibody reactivity against parasite proteins using Western Blot analysis have been shown (Fig. 2).

Levels of parasite-specific IgG antibodies were determined in plasma from four groups of individuals who differ in their level of exposure to malaria (Figure 1). Levels of parasite-specific IgG antibodies were similar in adult Liberians clinically immune to malaria and Ghanaian children in the age-groups 0-5 and 6-10, but were significantly higher than in non-immune Danish travellers (u-test, P < 0.001). This result is in agreement with the observation that the quantity of anti-parasite total IgG alone cannot explain naturally acquired immunity to *P. falciparum *asexual blood forms [17]. In order to further examine whether the IgG subclass distribution could play a role for the delayed acquisition of protective immunity, the parasite-specific IgG subclass profile in the above mentioned groups of individuals was characterized.

### Profiling of IgG subclass responses by immunoblot analysis of PEMS

Purified PEMS preparation was used as a source of proteins expressed in mature schizonts. Microscopic examination of the PEMS preparation showed merozoite clusters surrounded by the PVM (data not shown). Proteins extracted from the PEMS preparation were separated by SDS-PAGE and subjected to immunoblot analysis.

**Figure 2:**
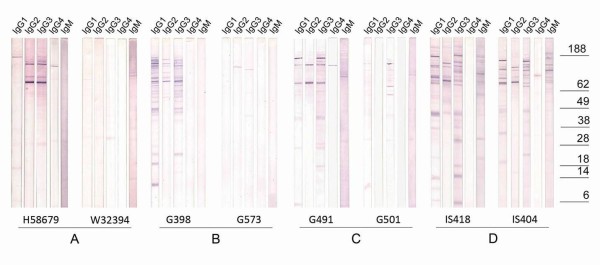
**Western blot analysis of IgG subclass responses**. Asexual blood-stage F32 parasite proteins (PEMS preparation, 100 μg) were separated by SDS-PAGE and blotted onto nitrocellulose membranes. Membranes were cut in vertical strips, blocked and incubated with individual plasma from A) Danish travelers who have suffered a single episode of *P. falciparum *malaria (n = 7), B) Ghanaian children age 0-5 (n = 8), C) Ghanaian children age 6-10 (n = 6), and D) clinically immune Liberian adults (n = 12). Antibody responses were revealed with monoclonal antibodies directed to human IgG1, IgG2, IgG3, IgG4 and IgM and visualized with AP-conjugated anti-mouse antibodies. For each of the different groups (A to D) reactivity of two representative individual plasma samples are shown, where the one to the left was seen to have high IgG titers determined by ELISA against late-stage parasite extract, whereas the one to the right had low IgG titers in the same assay. Western blots were performed with different SDS-PAGE gels, all loaded with the same protein preparation (100 μg) and run under identical electrophoretic parameters. The strips shown originate from a single blot.

Representative immunoblots displaying profiles of different antibody isotypes reactivity are shown in Figure 2. Both IgG4 and IgM isotypes were found to display weak and infrequent reactivity against parasite proteins in all groups. Amongst the remaining antibody isotypes, the IgG2 reactivity profile displayed a consistent pattern for nearly all plasma samples showing reactivity against a few high-molecular weight proteins with one protein of approximately 70-kDa being particularly strongly recognized by the majority of samples (Group A, 2/7; Group B 5/7; Group C, 4/8; Group D, 12/12). In contrast, the IgG1 and IgG3 band profiles differed greatly between plasma samples from different exposure groups (Figure 2A-D, lane 1 and 3). Another notable observation was the tendency for a broader IgG1 and IgG3 reactivity with increased exposure. As shown in Table 1, the number of IgG1 reactive bands below 70-kDa was significantly greater in clinically immune Liberians compared to non-immune Danish travellers (Wilcoxons rank sum test, P = 0.009). The difference between groups was even more pronounced for the IgG3 reactivity with a significant difference between adult Liberians and young Ghanaian children (Wilcoxons rank sum test, P = 0.006) and non-immune Danish travellers (Wilcoxons rank sum test, P = 0.010). In general, Danish travellers with a single malaria episode reacted against a few high-molecular weight antigens, whereas those from Liberian adults recognized a larger number of both high- and low-molecular weight antigens (compare Figure 2A and 2D and Table 1). Plasma samples from the Ghanaian children appeared to recognize an intermediate number of bands (Figure 2B and 2C and Table 1). Thus, increased exposure to *P. falciparum *malaria resulted in increased antibody reactivity against wider range of low-molecular weight parasite antigens.

**Table 1:** Differential immunoblot pattern of low-molecular weight (< 70 kDa) parasite antigens detected by cytophilic antibodies

Plasma samples		Number of bands detected (Median and interquartile range)	
Groups	Composition	IgG1	IgG3
A	Adult Danish travelers	5.0 (4.0-6.0)^c^	6.0 (3.0 - 8.0)^f^
B	Ghanaian children (0-5 yr)	5.5 (2.0-8.0)^b^	4.0 (3.0 5.0)^e^
C	Ghanaian children (6-10 yr)	6.5 (6.0-8.0)^a^	8.5 (4.5 - 13.5)^d^
D	Adult Liberians	9.0 (6.5-12.5)	14 (10.0 - 15.0)

Comparing IgG1 reactivity: ^a ^D vs. C, P = 0.121, D vs. B^b^, P = 0.094, and D vs. A^c^, P = 0.009. Comparing IgG3 reactivity: ^d ^D vs. C, P = 0.157, D vs. B^e^, P = 0.006, and D vs. A^f^, P = 0.010. P-values are based on Wilcoxons rank sum test with correction for ties.

## Discussion

Naturally acquired immunity against malaria is highly prevalent in adults residing in malaria endemic regions. It is the strongest known resistance against severe clinical malaria, and it develops over a long period with repeated infections. Passive transfer of purified IgG from hyperimmune sera to malaria patients has demonstrated that IgG mediates anti-parasite activity and protection against malaria. However, the mechanisms involved in the acquisition of naturally acquired immunity and the targets of the protective IgG have not been completely elucidated. This report demonstrates a differential parasite antigen reactivity of IgG responses acquired against *P. falciparum *asexual blood stage antigens at different exposure levels with a particular focus on parasite antigens expressed in mature schizonts. Using parasite PEMS preparation as the source of antigens, antigenic profiling of the naturally acquired IgG has been carried out using ELISA and Western Blot analysis of parasite antigens separated by SDS PAGE. Conventionally, the approaches widely used to study host immune response against malaria antigens have relied on the use of recombinant proteins or synthetic peptides usually representing subdomains of parasite proteins which could differ from their corresponding native parasite counter-parts in terms of their overall structures and post-translational modifications both of which could potentially alter the antigenic property of the target protein. Since the selection of antigenic targets of interest has traditionally been the first step for studying naturally acquired immunity against malaria, very few studies have attempted an unbiased pan-profiling of antibody reactivity against a broad range of parasite antigens [25]. Alternatively, other studies have attempted to profile antibody reactivity against the whole parasite extract primarily through ELISA [23,26], an approach which does not yield information regarding the molecular characteristics of the specific antigens recognized by the sera samples. Recently, protein microarray approaches have been developed which enable antigenic analysis against wider range of antigens, but still this technique relies on use of recombinant proteins or synthetic peptides and may suffer from the above listed limitations [27-29]. The present approach of unbiased profiling of naturally acquired antibodies against parasite antigens using native parasite extract has obvious advantages over the use of recombinant proteins or synthetic peptides. As the Western blot method involves denaturing and reducing the antigens the responses reported here might preferentially be against denatured antigens, whereby reactivity against some antigens characterized by having three-dimensional epitopes could have been missed. However, a recent study on the antibody response against *Schistosoma mansoni *showed that the antigenic profiling was the same whether antigens had been denatured and reduced or not [30]. Other advantages of using proteins from the parasite extract over recombinant proteins or peptides would include covering all linear epitopes over the full-length protein together with all the post-translational modifications which could affect antigenic properties [31].

The analysis of total anti-parasite IgG in individuals subject to different levels of exposure revealed unexpectedly high levels of anti-parasite IgG in the Danish travellers' plasma after a single episode of clinical malaria. Though polyclonal activation of host B-cell response has been suggested to be mediated by certain parasite antigens [32], this does not appear to explain the profile of the primary attack IgG subclass specific reactivity observed in the Western blot analysis. Primary attack plasma samples showed selective and restricted reactivity against high molecular weight parasite antigens with IgG1, IgG2 and IgG3 responses, but very little IgG4 and IgM reactivity, indicating activation of selected B-cell clones rather than polyclonal activation. It is interesting to note that the pattern of IgG2 reactivity developed against high molecular weight antigens as a result of primary attack is also observed in plasma samples collected from Ghanaian children and Liberian hyperimmune individuals with multiple attacks. Additionally, increasing exposure leads to a broader repertoire of *P. falciparum *reactive IgGs with the highest number of antigens being recognized by plasma from clinically immune Liberian adults. A degree of antigenic influence on subclass specificity is particularly evident for the IgG1 and IgG3 subclasses, with some bands being recognized predominantly by IgG1 or IgG3 whilst others are recognized by both classes. In contrast, the IgG2 antibody response seems relatively independent of host age and exposure to *P. falciparum*. It appears that the antigens recognized by IgG2 are few and in most cases of high molecular weight (> 70 kDa). One antigen of approximately 70 kDa was found to be particularly strongly recognized by majority of the plasma samples from all exposure groups. The apparently low prevalence of IgG2 antibodies to some malaria antigens is in agreement with many previous reports [6,33-35]. However, the observation that the IgG2 response in general seems independent of age and exposure is inconsistent with the prevailing hypothesis that the initial antibody response against *P. falciparum *is dominated by non-cytophilic antibodies (IgG2/IgG4 and IgM), and that this response gradually develops into a more protective response dominated by cytophilic IgG1 and IgG3 antibodies [17]. The data presented here suggests that the anti-*P. falciparum *IgG2 response is directed against a few high-molecular weight antigens and that the age-dependent IgG1 and/or IgG3 responses observed against several low-molecular weight proteins are not preceded by corresponding IgG2 responses.

This observation suggests that clonal B-cell activation is age and exposure dependent. It seems that upon subsequent malaria attacks, additional B-cell clones are activated, which primarily generate IgG1/IgG3 responses and which may contribute to the gradual development of immunity. The immune response to a wide range of antigens is weak upon the first few exposures to malaria infections. It is likely that the parasite may produce immune-suppressive factors [36-38] resulting in poor immunogenicity of at least some malaria antigens. However, upon repeated exposures to malaria infection a widened range of parasite antigens react with the developed antibodies. It is hypothesized that a first step towards developing IgG reactivity to wide range of parasite antigens and thus clinical protection would be to develop effective neutralizing factors towards such parasite encoded immune suppressive factors. This could happen in combination with age dependent physiological changes [39].

Results from this study suggest that the progressive development of anti-parasite immunity in older children is not mediated by a general switch of malaria-specific antibodies from the non-cytophilic to the cytophilic subclasses. Taken together, the data suggests that the long time required to acquire clinical protection against *P. falciparum *malaria is not only related to isotype switching towards ADCI-effective antigens but also to a gradual development of IgG1 and IgG3 antibodies against some previously non-targeted antigens like the distinct low-molecular weight *P. falciparum *proteins detected in this study.

## Conclusion

The present study confirms and extends previous observations suggesting that the cytophilic anti-*P. falciparum *IgG subclass responses increase with age and exposure, however, subclass switching from pre-existing non-cytophilic antibody responses to cytophilic subclass may not be a general phenomenon.

## Competing interests

The authors declare that they have no competing interests

## Authors' contributions

BV, SS and MT conceived the study. CHO, KB, SLD, PSJ, LSV, DD, PH, MC, SOL and SS performed the laboratory work and the statistical analysis. SS and MT wrote the manuscript. All authors have read the manuscript and agree with its contents.

## Acknowledgements

We thank M. Paulli Andersen for technical assistance.
